# Developing an Intelligent Mobile Clinic—A Medical Vehicle for Improve Access to Healthcare in Remote Areas: Evidence From China

**DOI:** 10.2196/59103

**Published:** 2025-06-02

**Authors:** Xinlei Chen, Xufang Huang, Yimiao Xu, Jiabin Xu, Yanan Wang, Xinyue Ren, Xuebo Zhu, Xiaoge Xie, Yeqin Yang

**Affiliations:** 1 School of Nursing, Zhejiang Chinese Medical University, No. 548, Binwen Road, Binjiang District, Hangzhou, Zhejiang Province, China, 86 13758719218; 2 Department of Nursing, Lishui Central Hospital, Lishui, China; 3 School of Nursing, Wenzhou Medical University, Wenzhou, China; 4 School of Medical and Humanities Management, Wenzhou Medical University, Wenzhou, China

**Keywords:** primary health care, medical resource, telehealth, intelligent mobile clinic, China, remote area

## Abstract

Lishui, a mountainous city in Zhejiang Province, China, is characterized by extensive mountainous terrain and a dispersed population. To address this issue, Lishui has introduced the intelligent mobile clinic service. This model leverages 5G technology and integrates the benefits of mobile clinics and telehealth, tailored to the region’s geography and demographic characteristics. The intelligent mobile clinic uses real-time data analysis to deliver medical services effectively to remote mountainous areas. Medical personnel from the intelligent mobile clinic visit villages to provide in-person professional health services and facilitate residents’ access to higher-level hospital resources through telehealth. This model has received widespread praise and recognition from residents and has yielded significant outcomes. During 2018-2023, a total of 25,000 visits have been made, benefiting 648,000 individuals. The intelligent mobile clinic provides a valuable reference for enhancing health care access in similar regions.

## Introduction

The unequal distribution of medical resources is a global issue, particularly in remote areas where access to health care is severely limited.

Telehealth presents an effective solution to address this disparity in health care resources. Its primary aim is to enhance health care access by providing services to individuals who face barriers to obtaining care [1]. Initially, telehealth was employed to treat acute conditions, such as trauma or stroke [2]. As health care demands have risen, telehealth has expanded to include care for military personnel, prisoners, and rural populations [3]. While telehealth has significant potential, its development depends on conditions such as network infrastructure and coverage. Mobile clinics provide an additional solution to the unequal distribution of health care resources. Mobile clinics are mobile health care units, typically modified ambulances, designed to deliver services to both urban and rural communities [4]. This flexible health care model overcomes both time and geographical challenges, bringing medical services directly to remote areas. However, the limited space in the mobile clinic restricts the number of staff and equipment available, making it difficult to meet the diverse health care needs of residents [5].

In Lishui City, Zhejiang Province, China, an innovative integration of telehealth and mobile clinics has resulted in the creation of the intelligent mobile clinic. This model effectively combines the strengths of both telehealth and mobile clinics to address the health care needs of those in mountainous regions.

In this study, we introduce an innovative medical model for improving medical services in remote mountainous areas in Lishui City, Zhejiang Province, China. We will use the application, effectiveness and difficulties of the intelligent mobile clinic model in remote mountainous areas of Lishui City to provide reference experience for other remote areas to improve the uneven medical resources and provide high-quality medical services.

## Health Care Inequities and Telehealth Innovations in Rural China

China is a predominantly agricultural country with a large rural population. As of 2022, the rural population in China reached 491.04 million, accounting for 34.78% of the total population [6]. Residents in remote areas face difficulties accessing health care due to factors such as economic constraints, distance, and time limitations [7-9]. For example, they have to spend a whole day seeking medical treatment, and some people may not even know that they are sick until their condition is very serious or has reached an advanced stage [10].

In response, the development of rural health care and medical systems has been the core of the new health care reform launched by China since 2009, which aimed at narrowing the urban-rural and regional disparities [11]. However, despite government efforts to implement medical reforms, there are still issues such as disparity distribution of health resources, a shortage of medical personnel, and inefficiencies in medical services [11,12]. Recognizing these challenges, the Chinese government has introduced various methods. One such method is the use of telehealth. Previous research has confirmed that telehealth can save residents time and costs in seeking medical treatment and promote the management of chronic diseases [13,14]. However, telehealth has not fully filled the health gap in rural areas, mainly due to young people flocking to urban centeres in search of better opportunities. This migration has left rural areas with a population dominated by older persons who face significant challenges in accessing and using e-health resources [15]. Consequently, these barriers prevent elderly residents from fully benefiting from telehealth services, exacerbating the health care disparities between rural and urban areas.

To address these challenges, China has been exploring innovative telehealth models that better suit the country’s unique circumstances in recent years.

## Intelligent Mobile Clinic in Lishui, China

A notable example is Lishui City, where a new solution has been introduced to address the specific health care needs of its rural population.

Lishui City is located in the mountainous region of southwest Zhejiang Province, where rugged mountains and hills dominate, with mountainous areas covering around 90% of the total land area. The region is home to more than 250 mountain villages, most of which are situated over 80 kilometers from the town center. Despite the local government’s efforts to improve health care services, the spread-out population and small village sizes make it difficult for health care to reach every village, failing to meet the basic health care needs of the residents. As a result, residents often face long journeys, taking an entire day to reach a health care facility.

In Lishui’s mountainous areas, as is the case in many rural parts of China, the population is largely elderly. These individuals often face additional challenges such as illiteracy, difficulty speaking Mandarin, and an inability to use smartphones or other smart devices. Although telehealth has significant potential to improve health care access, it struggles to address these issues in Lishui’s rural context. As a solution, the government of Lishui City has developed the intelligent mobile clinic, which delivers more accessible and effective health care services to the rural population. The intelligent mobile clinic represents an innovative response to the distinct needs of Lishui’s residents. The next section will provide a detailed introduction to the intelligent mobile clinic.

## The Intelligent Mobile Clinic: An Innovation Solution for Remote Area Health Care

**Figure 1.**
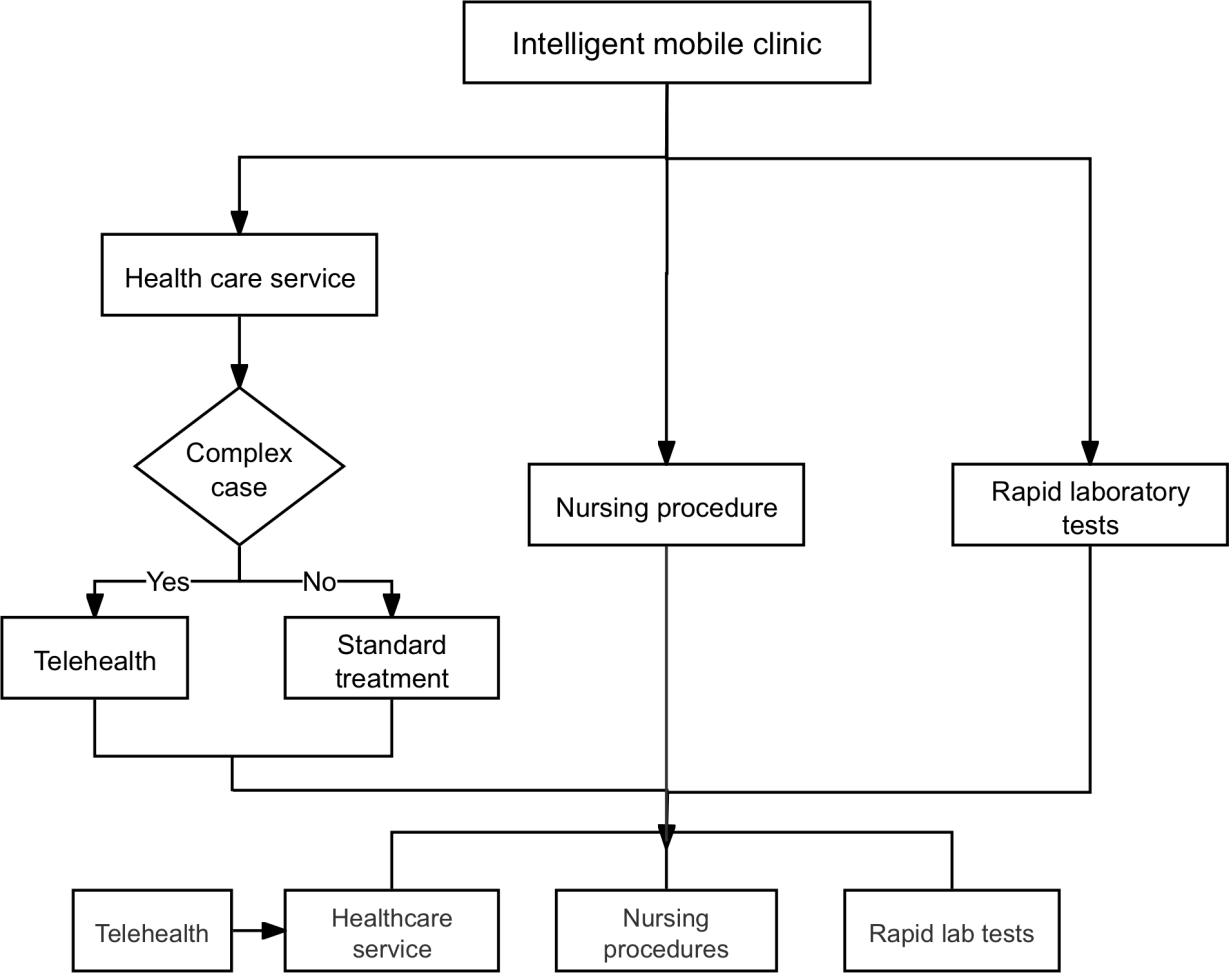
Flowchart of the intelligent mobile clinic health care service for residents.

The intelligent mobile clinic offers an innovative solution by combining mobile clinics, telehealth, 5G technology, and big data to tackle the unequal distribution of medical resources in remote areas. This model integrates several advanced technologies to provide high-quality and accessible health care services to people in remote regions (Figure 1).

The intelligent mobile clinic utilizes modified medical vehicles, equipped with essential diagnostic tools, to deliver health care services to even the most remote mountainous areas. These vehicles provide services such as routine health check-ups and rapid laboratory tests. When the doctor encounters cases that surpass their expertise, the telehealth system connects them with specialists at higher-level hospitals. This real-time consultation enables more accurate diagnoses and effective treatments, even in distant locations.

The roles of 5G and big data are pivotal in supporting the intelligent mobile clinic. 5G technology ensures smooth communication and the real-time transfer of medical data, while big data helps gather and analyze health information to refine services, monitor regional health trends, and improve the distribution of medical resources.

### Mobile Clinic Services in the Intelligent Mobile Clinic

The intelligent mobile clinic provides medical services to residents in remote areas. The mobile medical vehicles of the intelligent mobile clinic arrive at mountain villages at fixed times every month to provide medical services to residents.

Doctors provide direct consultations, perform routine tests, diagnose common diseases, and prescribe medication when needed. If a resident’s medical needs exceed the available services, doctors can consult experts at senior hospitals through telemedicine systems to ensure that residents receive specialized care. The nurse administers injections and performs dressing changes. Medical technicians perform rapid laboratory tests such as blood pressure monitoring, blood sugar tests, and electrocardiograms to assist in diagnosis. To improve communication with elderly people who are not fluent in Mandarin, the intelligent mobile clinic recruited local volunteers who are fluent in Mandarin and regional dialects. These volunteers play a vital role in maintaining order and helping elderly patients onto medical vehicles. It is worth noting that in order to ensure that patients who are bedridden at home can also enjoy medical care services, doctors provide medical services for patients at home, replace drainage tubes, urine bags, etc.

### Telehealth in the Intelligent Mobile Clinic

When medical vehicles are unable to fully meet the health care needs of residents, the intelligent mobile clinic doctors use telemedicine to consult experts in senior hospitals. Through telemedicine devices, doctors can transmit real-time health data to specialists, which ensures that residents have access to vital health care services and receive timely medical attention.

In addition, the 5G network improves the efficiency of telemedicine within the intelligent mobile clinic. It provides a reliable, stable link for online consultations, overcomes geographical barriers, and gives mountain residents access to the same medical services and resources as urban residents. As a result, the intelligent mobile clinic has significantly improved the accessibility and quality of health care services in remote areas.

### Big Data Support in the Intelligent Mobile Clinic

Big data technology is the key technology for the intelligent mobile clinic to achieve precision medicine. Relying on 5G network technology, the intelligent mobile clinic transmits the relevant data of 50 medical vehicles in real time, including GPS positioning information, diagnosis and treatment records, and disease conditions of patients in various regions to the Lishui Health Information Platform and terminal personnel can form regional health information self-portraits according to the information transmitted by all medical vehicles through big data. It is worth noting that the medical staff on board each medical diagnosis and treatment vehicle can only view the relevant diagnosis and treatment information in their own area of responsibility to protect the privacy of patients.

### Extra Functions of the Intelligent Mobile Clinic

The intelligent mobile clinic has taken into account the varied medical needs of villagers, including the delivery of medications to mountainous areas. Due to certain restrictions, the intelligent mobile clinic is not able to carry all types of medicine. When specific medications are needed, the doctor will write a prescription and request confirmation from higher-level doctors via the network. Upon approval, the medication is prepared by the higher-level hospital and sent to the villagers free of charge through express delivery.

Furthermore, to better meet the medical needs of the villagers, the intelligent mobile clinic also functions as a first-line emergency responder. In emergency situations in remote villages, such as bleeding injuries or drowning incidents, villagers can contact the emergency hotline. The dispatcher will then assign the nearest medical vehicle from the local health center to respond based on the villager’s location.

### Routine Operation and Management

The intelligent mobile clinic collects and addresses the medical needs of the villagers through multiple channels: most villagers line up at the medical site on a fixed date each month. Villagers who have the ability to use mobile phones can access the feedback module by logging into the interface to submit medical requests, while those who do not have access to phones can make requests through the hotline, and the information is entered into the system by staff. Local community hospitals or health centers flexibly adjust the allocation of medical staff and drug supplies based on these real-time data.

**Figure 2.**
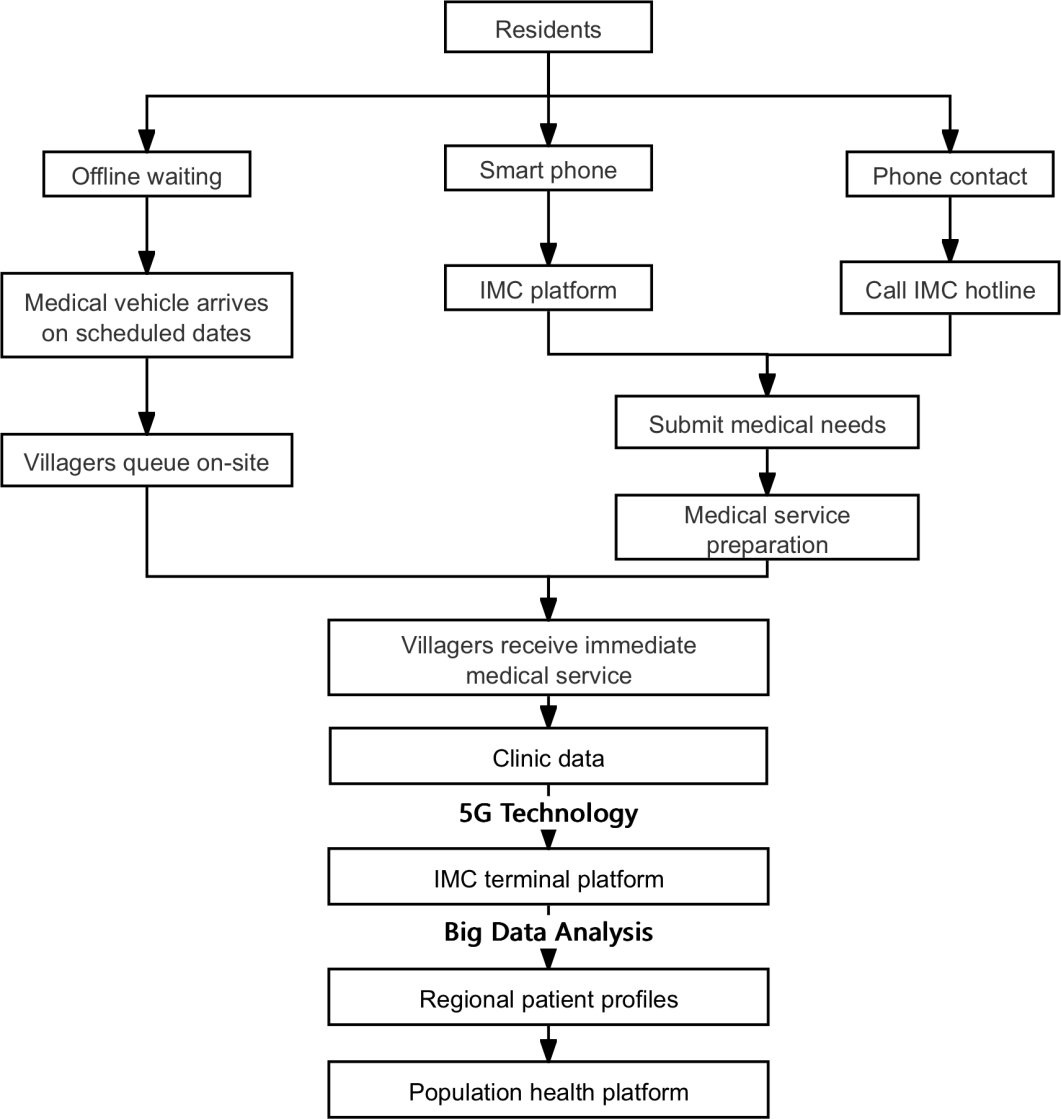
Flowchart of the intelligent mobile clinic (IMC) routine operation and management.

In order to achieve efficient service management, the intelligent mobile clinic has created a comprehensive database by integrating vehicle information, medical personnel files, service data, GPS coordinates, and digital maps. The management platform transmits diagnostic records, treatment processes, and vehicle operating status in the diagnosis and treatment vehicle to the terminal platform in real time through 5G technology; and generates regional health information portraits through big data analysis. As regulators, the government and health departments can remotely verify the number of medical personnel, vehicle positioning, and diagnosis and treatment process with the help of monitoring cameras in the diagnosis and treatment vehicle to ensure the quality of service. In the design of the system, the information security mechanism is especially strengthened, and regional data rights management is implemented, that is, doctors in each region can only access the patient information in their jurisdiction, and the privacy security of villagers is protected from the technical level (Figure 2).

## Comparing the Intelligent Mobile Clinic With the Mobile Clinic and Internet Hospitals

### Overview

Several models currently exist to provide primary health care services to residents in remote areas. Mobile clinics represent an innovative health care service model that delivers flexible medical services to targeted populations through a customized motor vehicle [16]. Internet hospitals are platforms where registered doctors provide consultations and treatments via the internet, primarily addressing common or chronic health conditions [17]. The rapid development of internet hospitals in China is expected to improve the availability of medical resources and reduce the cost of telehealth services [18].

Despite their benefits, both models have certain limitations. Mobile clinics are restricted by the limited space in medical vehicles, which limits the amount of equipment, medicines, and medical personnel that can be carried on each visit [19]. As a result, in unexpected situations, the medical needs of residents may not be fully met. Internet hospitals services are faced with multiple practical challenges when used for primary medical coverage. First, internet hospitals have high requirements for mobile devices and lack communication equipment in remote mountainous areas [20,21], which makes it difficult for people to effectively enjoy telehealth services [22,23]. Second, the physical isolation between doctors and patients in the remote diagnosis and treatment mode makes it impossible for doctors to perform basic physical examinations for patients, which may affect the completeness and accuracy of the clinical diagnosis. Third, after using intelligent terminals to complete online consultation, elderly patients also need to go through the drug logistics and distribution link, which increases the time cost compared with traditional medical treatment [24].

**Figure 3.**
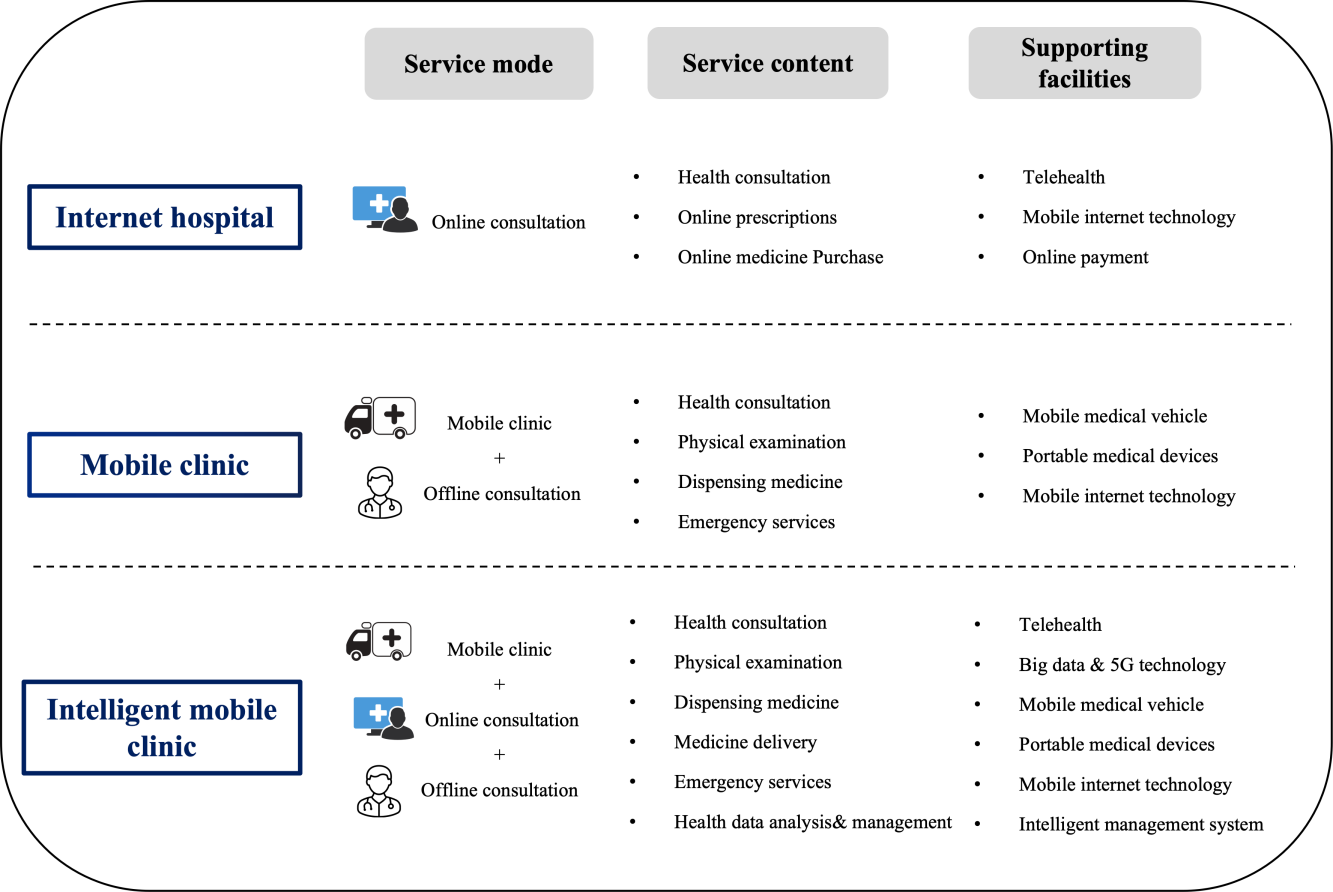
Comparison of 3 medical models.

In contrast to mobile clinics and internet hospitals, the intelligent mobile clinic integrates the benefits of both online and offline services. Local doctors provide in-person consultations and treatments. When additional expertise is needed, the telehealth system connects them with specialists from higher-level hospitals, offering effective support and guidance. Additionally, the convenience of these services results in high patient satisfaction. This combined approach allows the intelligent mobile clinic to address the limitations of both mobile clinics and internet hospitals, delivering a more comprehensive and accessible health care solution for remote populations (Figure 3).

To evaluate the real user experience of the intelligent mobile clinic, a telephone follow-up was conducted with residents in January 2024. The research team focused on villagers from Bihu Town, Lishui City, conducting qualitative interviews with them (Table 1). The interviews collected data on basic user characteristics, home addresses, and their evaluations of the intelligent mobile clinic services. The findings reveal that the respondents gave high ratings to the intelligent mobile clinic, highlighting that it significantly meets their health care needs.

**Table 1.** Qualitative interviews of the user experience of the intelligent mobile clinic (IMC).

Interviewee #	Age, y	Gender	Disease	Job	Interview content
1	50	Female	Diabetes	Run a grocery store	I have diabetes and need to take my medication regularly. The medical vehicle visits our village, which makes it very convenient for me to get my medication. I can receive it immediately without needing to go to the hospital.
2	52	Male	Disability,hypertension	Farmer	I am disabled and have high blood pressure, so I must take medication every day. Given my condition, I cannot travel to an external hospital by myself. The medical vehicle visits our village once a week, which is very helpful.
3	57	Female	Hypertension,hypothyroidism	Farmer	The IMC is a great help. We live in a rural area with no public transportation, and the medical vehicle comes every week, mainly to visit patients with chronic diseases. For more serious cases, the doctor will refer patients to a higher-level hospital for treatment.
4	56	Female	Hypertension	Farmer	The IMC is well-received by the villagers. They come once a week and bring fresh medicines. Since we do not have a clinic in our village, we have to travel to nearby towns or urban areas for medical care. This has led many people in the village to wait in line for the mobile vehicle to arrive.
5	50	Female	Hyperlipidemia	Farmer	It is far more convenient to see a doctor and get medication, as there is no need to wait in long lines as in traditional hospitals. The outpatient fee at the mobile clinic in the village is just 5 RMB (US $0.7).

### Ethical Considerations

This work adheres to ethical guidelines for scholarly commentary. All referenced studies comply with institutional ethics standards. The original research cited in this article was approved by the Zhejiang Chinese Medical University Institutional Review Board (IRB#20250220-1). All the patients provided informed consent for use of their data; all the data were de-identified.

## Results

From 2018 to the end of 2023, the intelligent mobile clinic had a total of 50 mobile medical vehicles, a total of 18,000 visits, 100 years of travel within the area of 10,000 kilometers, a total of 25,000 village medical services, 33,000 medical personnel, a total of 657,000 services, covering 758 remote villages without medical facilities (Table 2). In addition, the average emergency response time in mountainous areas reduced from 34 minutes to 14 minutes. According to data from the Lishui government, the goal of mountainous residents to have “shorter medical treatment times and the ability to address minor illnesses within the village” has increasingly become a reality.

**Table 2.** Characteristics of patients who used the intelligent mobile clinic from 2021 to 2023.

Characteristics, n (%)	2021	2022	2023	Total
Total users	91,287 (10.38)	130,445 (14.84)	656,998 (74.78)	878,730
Number of medical vehicle rounds	2113 (11.60)	5550 (30.47)	10,549 (57.93)	18,212
Number of village visits	4942 (19.11)	5426 (20.98)	15,492 (59.91)	25,860
Number of medical staff	3043 (9.01)	9486 (28.08)	21,257 (62.91)	33,786
Type of medical service				
Diagnosis and treatment	9646 (3.54)	46,465 (17.05)	21,6471 (79.41)	272,582
Traditional Chinese medicine	4765 (4.76)	19,222 (19.20)	76,115 (76.04)	100,102
Prescription	700 (0.35)	36,553 (18.17)	164,591 (81.48)	201,144
Laboratory testing	386 (1.30)	1821 (6.15)	27,386 (92.55)	29,593
Chronic disease follow-up	68,790 (25.71)	26,384 (9.86)	172,435 (64.43)	267,609

## Discussion

The emergence of the intelligent mobile clinic has effectively alleviated the unequal distribution of medical resources in remote mountainous areas of Lishui City, and increasing numbers of villagers can get timely treatment. As noted earlier, the population in the remote areas of Lishui is spread across the mountains, making it difficult for residents to access medical resources from urban areas. Building hospitals in these regions would serve only a small portion of the population, potentially resulting in a waste of resources. Additionally, many residents in these mountainous areas are elderly and face challenges such as illiteracy, an inability to speak Mandarin, or limited access to the internet. These factors mean that internet hospitals and mobile clinics may not fully meet their needs. Each region has its own unique circumstances, and local governments must carefully assess these conditions and implement appropriate measures to provide suitable services for residents, such as capsule clinics in Ningbo or internet hospitals in Guangzhou. The intelligent mobile clinic is a new health care service model that integrates the advantages of telehealth and mobile clinics, developed by the local government to address the specific conditions of the area. With the guidance of big data, the intelligent mobile clinic is able to deliver accurate health care services to residents in remote mountainous regions. Medical staff from the intelligent mobile clinic also help elderly residents communicate with specialists through the internet.

## Conclusion and Limitations

The intelligent mobile clinic presents an innovative and effective solution for promoting the equal distribution of medical resources in remote areas. By combining the benefits of telehealth and mobile clinics, the intelligent mobile clinic tackles the health care challenges in the mountainous regions of Lishui. The success of the intelligent mobile clinic in Lishui offers a valuable example for addressing similar issues in other remote areas of China and even in countries facing comparable challenges.

Nonetheless, each region must carefully evaluate its specific circumstances and develop suitable approaches to meet the health care needs of its residents, ensuring a balanced distribution of medical resources across various areas.
